# Imaging findings of the primary mucoepidermoid carcinoma of the breast

**DOI:** 10.1002/ccr3.5449

**Published:** 2022-02-11

**Authors:** Guiwu Chen, Wenqin Liu, Xiaomin Liao, Fenfen Yu, Yuhuan Xie

**Affiliations:** ^1^ Department of Ultrasound Affiliated Dongguan Hospital, Southern Medical University Dongguan People's Hospital Dongguan China; ^2^ Department of Pathology Affiliated Dongguan Hospital, Southern Medical University Dongguan People's Hospital Dongguan China; ^3^ Department of Radiology Affiliated Dongguan Hospital, Southern Medical University Dongguan People's Hospital Dongguan China

**Keywords:** breast tumor, magnetic resonance imaging, mammography, mucoepidermoid carcinoma, ultrasonography

## Abstract

Mucoepidermoid carcinoma (MEC) is an invasive tumor that has been reported in many organs, such as the salivary glands, lungs, esophagus, and thymus; however, it rarely affects the breast. Here, we report a case of primary breast MEC with imaging, including mammography, ultrasonography, and magnetic resonance imaging.

## Graphical abstract

Primary mucoepidermoid carcinoma (MEC) of the breast is a rare malignant tumor, which is limited to imaging, especially ultrasonography. We present the imaging of primary breast MEC, including mammography, ultrasonography, and magnetic resonance imaging.

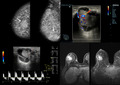

A 38‐year‐old woman with a history of breast masses for 1 year presented to our hospital with a mass rapidly grown in 1 week. On physical examination, the mass was palpated in the lower outer quadrant of the right breast, which felt pliable but strong, was well‐defined and movable. Before being admitted to the hospital, mammography was performed, which revealed a well‐defined huge mass with a partly‐lobulated boundary (Figure 1).

**FIGURE 1:**
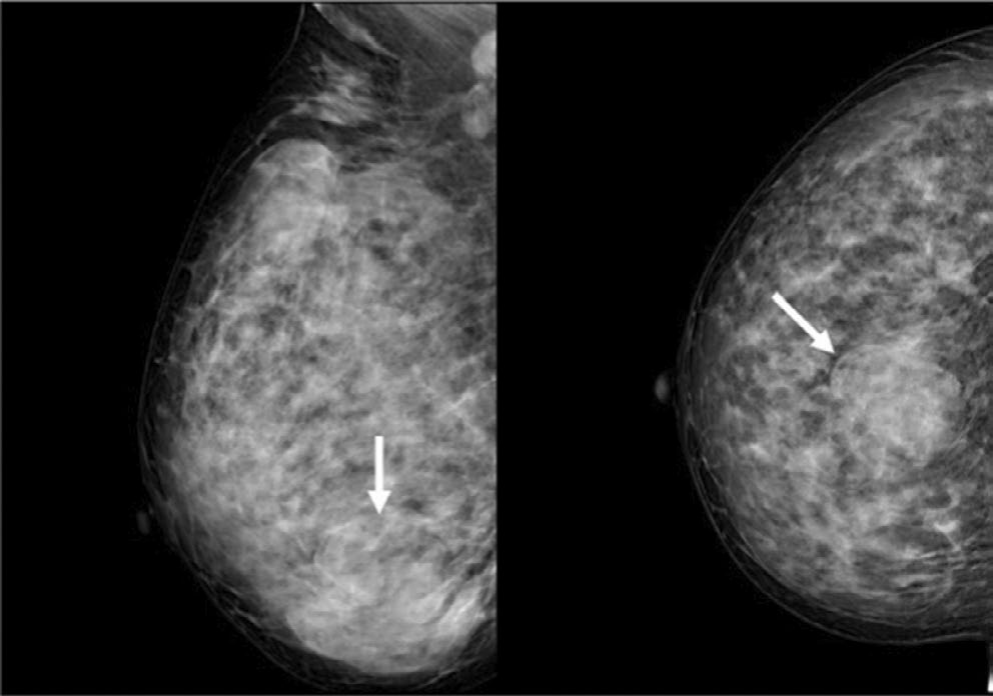
Mammography in the case of primary breast mucoepidermoid carcinoma showed the biggest mass was a well‐defined mass with a partly‐lobulated boundary as seen on the axial and coronal plane views

Initially, breast ultrasound examinations were performed. Multiple cystic breast masses bilaterally were observed; most of them were oval, anechoic, well‐defined, and thin‐walled. However, the biggest mass was some solid tissue within the septa‐divided cystic spaces with rich blood flow signals and high resistance (Figure 2). No lymph node metastasis was found in the bilateral axillary nodes.

**FIGURE 2:**
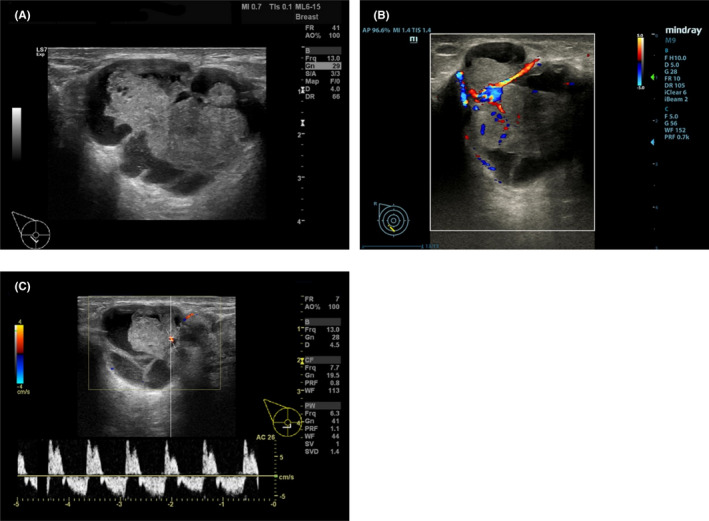
Ultrasonography of primary breast mucoepidermoid carcinoma. (A) Gray‐scale ultrasound showed the mass was irregular, lobulated, and well‐defined. (B) Color Doppler ultrasound showed the solid tissue with rich blood flow signals. (C) Pulsed‐wave Doppler ultrasound showed the blood flow had high resistance

Afterward, magnetic resonance imaging (MRI) of the breast was performed, which showed different signals at the solid tissue and peripherally high signals at the mass on T1‐weighted and T2‐weighted imaging. Additionally, it was observed that the lateral thoracic artery of the right breast was thicker (Figure 3).

**FIGURE 3:**
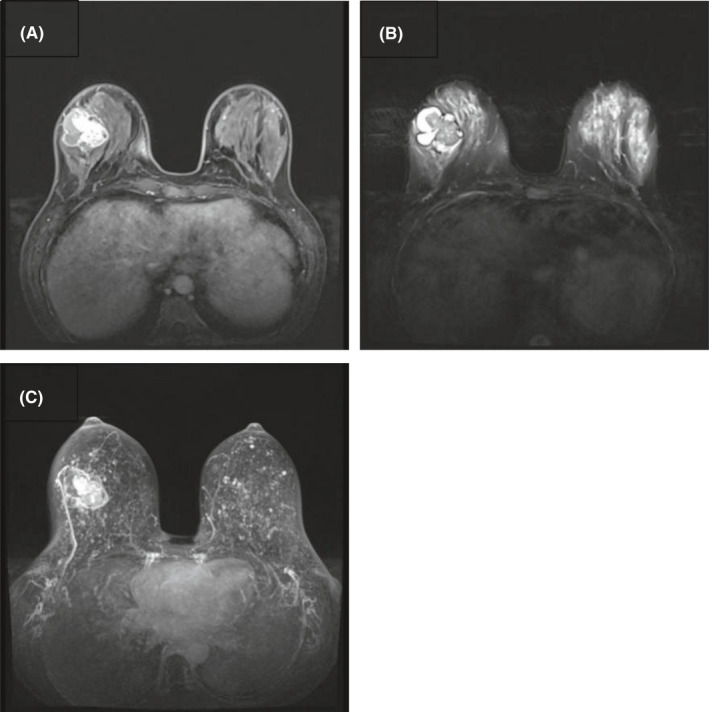
Magnetic resonance imaging of primary breast mucoepidermoid carcinoma. (A) T1‐weighted imaging showed the solid tissue had an equal‐intensity signal with a high‐intensity signal at the periphery of the mass. (B) T2‐weighted imaging showed the solid tissue had a low‐intensity signal with a high signal at the periphery of the mass. (C) Maximum intensity projection showed the lateral thoracic artery of the right breast was thicker than the left breast

Eventually, the patient underwent surgical resection of the biggest mass, and a diagnosis of primary low‐grade MEC was confirmed. No recurrence and metastasis were reported during the 6‐months follow‐up postoperatively.

Most of the case reports have insufficient imaging. Only a few cases of breast MEC mammography have been reported which described an unclear mass with or without accumulation of calcific deposits. Likewise, limited ultrasound images of primary breast MEC show a complex cystic or solid mass with a rough or regular surface.^1^, ^2^ When ultrasound is unavailable or inconclusive, MRI offers more diagnostic information to confirm the characteristics of the mass, including its components and composition; however, the evidence is still too deficient to characterize the findings.

## CONFLICT OF INTEREST

The authors declare that there is no conflict of interest.

## AUTHOR CONTRIBUTION

Guiwu Chen wrote the original draft of this clinical image and made subsequent revisions. Wenqin Liu participated in the ultrasound images analysis and interpretation. Xiaomin Liao participated in the pathology images analysis and interpretation. Fenfen Yu participated in the mammography and MRI images analysis and interpretation. Yuhuan Xie assisted in the revision and supervised the overall production of this report.

## ETHICAL APPROVAL

The corresponding author had the written consent of the patient to use the data for publication.

## CONSENT

The patient agreed to use his/her information and samples (including blood, urine, excrement, and excised tissue) for medical research for non‐commercial purposes under the premise of strict privacy protection.

## PERMISSION TO REPRODUCE MATERIAL FROM OTHER SOURCES

No material from other sources was applied.

## Data Availability

The data used to support the findings of this study are available from the corresponding author upon request.
